# User Experience Study of the Patient Monitoring Systems Based on Usability Testing and Eye Tracking

**DOI:** 10.3390/healthcare12242573

**Published:** 2024-12-20

**Authors:** Hyeonkyeong Choi, Wonseuk Jang

**Affiliations:** 1 Department of Medical Device Engineering and Management, Yonsei University College of Medicine, Seoul 06229, Republic of Korea; hyeonkyeong97@daum.net; 2 Medical Device Usability Research Center, Gangnam Severance Hospital, Yonsei University College of Medicine, Seoul 06230, Republic of Korea

**Keywords:** patient monitoring system, usability, Health-ITUES, SUS, eye tracking

## Abstract

Background/Objectives: The patient monitoring system is a critical tool commonly used in hospitals, making it essential to assess caregivers’ user requirements and satisfaction with its usability. In intensive care units (ICUs), the usability of these systems is closely linked to the work efficiency of key users, such as nurses, and directly impacts patient safety and treatment outcomes. This study evaluates the usability of patient monitoring systems in intensive care units (ICUs), focusing on user requirements and satisfaction among nurses, the primary users. Usability is directly linked to work efficiency and patient safety, with post-marketing surveillance (PMS) data from overseas, highlighting issues such as unrecognized alarms, leading to worsened patient conditions. Methods: This study involved 22 ICU nurses who had used the system for over a year, assessing usability through testing, satisfaction surveys, the Health-ITUES, and eye-tracking analysis. Results: The results showed a high success rate (94%) and positive satisfaction scores (4.15, SD = 0.88), with a Health-ITUES score of 4.13 (SD = 0.78). Eye tracking revealed that some functions, including alarms, were overlooked or not recognized. Conclusions: Recommendations include improving the interface for alarm messages and recording deletion functions to enhance user satisfaction and patient safety.

## 1. Introduction

### 1.1. Patient Monitoring System

Patient monitoring devices are among the most widely utilized tools in hospitals, essential for tracking and managing patients’ vital signs across various clinical environments [1]. These devices are especially crucial in intensive care units (ICUs) and operating rooms, where the continuous observation and the evaluation of patient conditions are paramount [1,2,3]. By providing real-time data on vital signs, patient monitoring devices help prevent deterioration in patient health and support timely medical interventions.

In critical care settings, the accuracy and reliability of these devices are vital [1]. Errors in parameter values or alarms can pose significant risks, making the usability of the user interface a critical factor [1,4]. A well-designed interface ensures that medical staff can quickly and accurately interpret data, which is crucial for effective patient management [2].

Despite advances in technology, the design of patient monitoring devices has remained largely unchanged for over two decades, with minimal alterations in layout and structure [1]. While usability evaluations have typically focused on main screens displaying waveforms and parameters or on user interfaces emphasizing trends and alarms, scenario-based usability testing has been notably absent in many studies [1,5]. Previous research has shown that usability evaluations, such as those conducted on GE monitoring devices, often emphasize setting alarms and trend parameters [5]. Although domestic hospitals predominantly use foreign-made devices due to their superior user interfaces and ease of use, this study aims to conduct a comprehensive usability evaluation of patient monitoring devices that have seen limited design changes. The goal is to assess and enhance these devices to improve user satisfaction in clinical settings.

**Figure 1:**
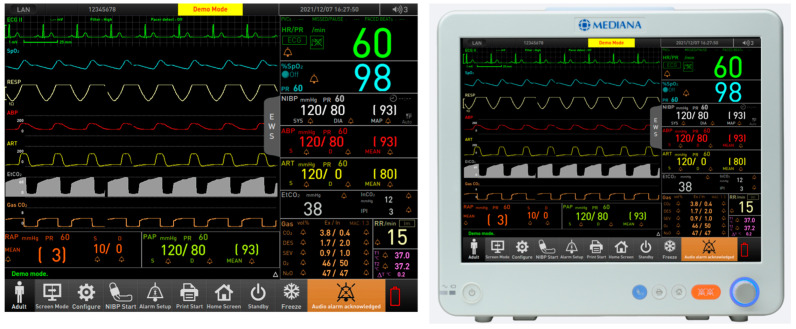
Patient monitoring system (M50).

Patient monitoring devices are typically organized into waveform and parameter areas, and they often include a menu button for navigation [1,6]. For example, as shown in Figure 1, the M50 Patient Monitor is equipped to provide a comprehensive array of measurements, including the electrocardiogram (ECG), heart rate (HR), non-invasive blood pressure (NIBP), oxygen saturation (SpO_2_), pulse rate, respiration rate, temperature, capnography (EtCO_2_ and InCO_2_), invasive blood pressure (IBP), multi-gas analysis, and bispectral index (BIS). Its 15-inch touch screen enhances the visibility and ease of use for medical personnel, while the built-in battery ensures functionality even during patient transport or power outages. These features collectively contribute to the device’s effectiveness in high-stake medical environments. The device’s graphical user interface enables the simultaneous display of four to seven waveforms within the waveform area.

### 1.2. Usability

Interest in designing medical devices with a focus on patient safety has been increasing since the 1990s [7,8,9]. It is crucial to ensure that medical devices are both safe and user-friendly [10,11,12]. While clinical requirements, human error, and patient safety are important factors, prioritizing user requirements is essential in the design process [7]. Medical devices are generally categorized into hospital devices and personal devices, each with distinct user profiles. Usability testing is critically important as it identifies usage errors through user interaction with the device interface and allows for analysis of error frequency and causes through interviews [8,10,11]. Although evaluating each user individually is time consuming, objective usability can be assessed based on the observed errors [7,8,10,11,12].

The “Content of Human Factors Information in Medical Device Marketing Submissions” guidance issued by the U.S. FDA in 2022 stipulates that the Human Factors Engineering (HFE) report must classify tasks according to their criticality at the time of marketing [13]. According to this guidance, critical tasks are those deemed essential for device design and are associated with the level of risk. To determine if a task is critical, it is necessary to identify and assess known usage errors both domestically and internationally, typically through investigations using the FDA’s Manufacturer and User Facility Device Experience (MAUDE) database.

A usability test is conducted throughout the entire lifecycle process of a medical device to evaluate how convenient the device is. It focuses on assessing the usability of the device in evaluation rather than comparing it with other similar devices to identify areas for improvements in the device’s design. A task analysis based on the device’s workflow is executed, and, during this process, the performance of the tasks is categorized into completed (C), completed with issues (CI), or not completed (NC). This approach allows for task success determination. Eye tracking can be used to analyze the gaze of participants to verify if their gaze correlated to task completion.

### 1.3. Eye Tracking

**Figure 2:**
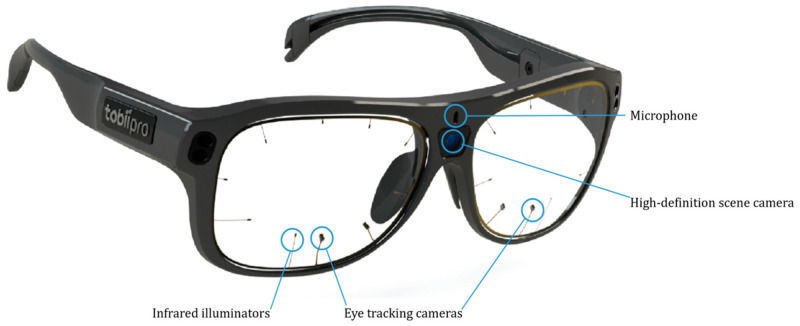
Tobbi Pro Glasses 3.

As shown in Figure 2, Eye tracking involves monitoring the gaze of a participant by analyzing the movement of the pupil via a camera [14,15,16,17,18]. Although research into eye tracking is expanding, its application for evaluating medical devices remains limited [18]. Since medical devices are used directly with patients, improving their usability is essential to ensure convenience and efficiency for medical staff and users. Eye tracking can play a significant role in enhancing this usability.

Eye trackers are generally categorized into two types: wearable and fixed [18]. Fixed eye trackers are advantageous for analyzing subjects whose eye movement is minimal or when the screen remains stationary. Conversely, wearable eye trackers are preferable when the device’s screen frequently moves or when there is significant movement by the target of eye tracking.

Eye tracking offers objective insights into user perception and is a valuable tool for evaluating usability. Key terms associated with eye tracking include fixation, saccades, heatmaps, and areas of interest (AOIs) [14]. Common eye-tracking metrics used in evaluations are dwell time, the number of fixations, fixation duration, the sequence, the time to first fixation, the revisit rate, and the hit ratio [14]. Among the indicators obtained through eye tracking, dwell time refers to the total duration of time spent focusing on a specific AOI, while the number of fixations indicates the frequency with which the gaze stops on a particular AOI within the recorded data [19,20,21]. Fixation duration represents the average time that the eye remains fixed on a specific AOI during a single fixation [22]. The sequence reflects the order of gaze movements, allowing for an analysis of the flow and pattern of attention directed towards specific information or stimuli [23]. Time to first fixation measures the time elapsed before the first fixation on a specific AOI, providing insight into attentional prioritization [20]. The revisit rate indicates the frequency with which attention returns to a given AOI, and the hit ratio serves as a measure of how successfully a specific task has been completed [20]. These indices provide critical data for assessing how users interact with medical devices and for identifying areas for improvement.

According to previous research, usability evaluations of user interfaces have typically relied on task completion assessments conducted through usability tests [14,16,24]. However, in practice, few studies have incorporated eye-tracking data to evaluate user interfaces during such tests [16,24]. In a previous study, the inclusion of eye-tracking data provided more objective results when performing usability tests, offering deeper insights into user interactions and supporting user interface improvements [24].

In a prior study evaluating the technical proficiency of orthopedic residents during hip arthroscopy training, indicators such as dwell time, fixation count, and saccade frequency were assessed [25]. Similarly, in the context of usability evaluation for syringes in intensive care settings, eye tracking was employed to analyze how nurses interacted with the attached labels [18,26]. Metrics such as dwell time, fixation count, and fixation duration were analyzed to evaluate the size and shape of the label’s text, aiming to minimize medication administration errors [26]. Visual adjustments were subsequently implemented to enhance usability and safety. Furthermore, eye-tracking technology was employed to evaluate the usability of three different intensive care ventilators by measuring task difficulty through variations in pupil diameter [24]. The comparative analysis of the ventilators’ usability underscored the efficacy of eye-tracking indicators in capturing user behavior and interactions with medical devices [24]. These findings highlighted the frequent application of dwell time, fixation count, and fixation duration as key metrics. The research emphasized the effectiveness of eye-tracking technology as a tool for evaluating user experience in medical device design, ultimately contributing to improved patient safety and satisfaction.

This approach allowed for a better understanding of user behavior that was not achievable through task completion metrics alone. Consequently, the integration of eye tracking into usability testing helped pinpoint specific design modifications necessary to optimize the overall user experience and enhance the usability of the interface. This study aimed to evaluate the usability of patient monitoring systems using eye-tracking technology to identify key areas for interface improvement.

## 2. Materials and Methods

### 2.1. Recruitment

The intended users of the patient monitoring system are nurses working in an intensive care unit. According to the IEC 62366 international standard, a formative evaluation requires the recruitment of at least 5 intended users, while a summative evaluation mandates a minimum of 15 participants. In this study, we adhered to the international standard, and, additionally, to meet the company’s internal requirement of 22 participants, we selected a total of 22 users for the usability evaluation. We recruited 22 nurses from Severance Hospital in South Korea. We recruited through recommendations from nurses. In this study, participants were selected based on their experience of using patient monitoring systems for more than one year and their employment in the ICU for at least one year. Nurses with less than one year of ICU experience were excluded from the evaluation. Since patient monitoring systems are primarily used in ICUs and the parameters and features vary across different ICU environments, nurses from various ICUs were recruited. We selected nurses from the Medical Intensive Care Unit (MICU), Surgical Intensive Care Unit (SICU), and Cardiac Care Unit (CCU) to represent the diversity of the intensive care unit (ICU) using the patient monitoring system. Additionally, because the patient monitoring system displays information in both English and Korean, only participants proficient in both languages were included in the evaluation. Also, this study complied with the Institutional Review Board of Yonsei University Health System, Gangnam Severance Hospital (No: 3-2023-0372).

### 2.2. Testing Procedure

To conduct usability testing, we developed a use scenario that reflects the workflow of patient monitoring systems, informed by post-marketing surveillance (PMS) data as reported to the FDA’s Manufacturer and User Facility Device Experience (MAUDE) database. We analyzed the PMS data concerning the appropriateness of use for these devices (product code: MHX) from 1 January 2021, to 30 June 2023. Through this analysis, we identified frequent events and potential risk factors associated with the use of patient monitoring systems.

The critical task selection criteria were established based on the severity of potential harm, as outlined in IEC 62366-1:2020, Clause 5.5, which pertains to the selection of use scenarios related to risk factors for overall assessment. Out of 4325 PMS records reviewed, 192 cases specifically related to the fitness for use of the MHX devices were examined. We identified and analyzed hazards such as alarm system failures, cable issues, and device drops, which were incorporated into the use scenarios. Tasks with a severity score of 3 or higher were designated as critical tasks. Additionally, alarm parameters were derived from alarm-related adverse events and used as evaluation metrics in the usability testing.

The usability test in this study was conducted with a total of 22 participants, each of whom completed the evaluation individually. Each participant followed the sequence guided by the moderator and completed the assessment accordingly. During the test, an observer in a separate observation room recorded the session on video and documented observations on a sheet. In total, the assessment involved one moderator, one observer, and one simulator operator.

**Figure 3:**
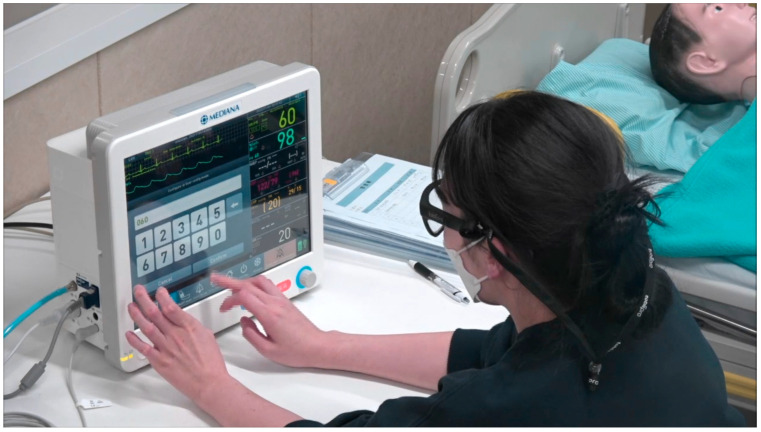
Participants wearing eye trackers while performing usability tests.

A moderator introduced the participants to the usability test and eye-tracking technology, obtained informed consent, and provided a 20 min training session on using the device. As shown in Figure 3, participants were instructed to inspect the lenses and complete a calibration step before wearing the eye-tracking glasses for the eye tracker evaluation. Due to variations in the location of the fovea, which is responsible for focusing the human eye and enabling the perception of all colors, calibration is essential to enhance the accuracy of eye tracking [27]. Among the various calibration methods, using calibration targets has been proven to provide the highest accuracy for eye-tracking data [27]. Calibration must be performed individually for each participant prior to initiating any evaluation. When utilizing the Tobii Pro Glasses 3, the calibration target should be positioned at a distance of approximately 50 to 100 cm from the participant [27]. The participant must then focus their gaze on the center of the target [27]. When the participant’s line of sight is accurately aligned, the gaze indicator will change from red to green, confirming successful calibration [27].

They were also informed that the glasses should not be removed during the evaluation and that the usability assessment must be conducted while wearing them. To evaluate usability, we assessed the time that participants spent fixating their gaze during the test tasks [28,29,30]. Gaze fixation time reflects the complexity of information processing or cognitive activities. Longer fixation times indicate greater difficulty in processing information or performing cognitive tasks, suggesting an increased workload [31]. Participant gaze data were collected using the Tobii Glasses 3 eye tracker (Tobii Technology, Danderyd, Sweden). We utilized the data analysis feature in a program called Tobii Pro Lab version 1.241. To generate the heatmap image, we employed the “Visualization Type and Settings” tool (specifically the Gaze Visualization Layers in versions 1.241 and later) within Tobii Pro Lab. We identified the point in time when the use error occurred to examine the corresponding heatmap.

Participants were given as much time as needed to interact with the device. To minimize the potential impact of the training on the evaluation, a 15 min interval was observed between the training and the evaluation. Following this interval, the participant wore the eye tracker for the usability evaluation.

Participants completed a usability test consisting of six scenarios, which included a total of 30 tasks designed for nurses. The six scenarios were as follows: remote control, registering patients, connecting cables, alarm, data trend review, and standby mode. The specific tasks associated with each scenario are detailed in Table 1.

**Figure 4:**
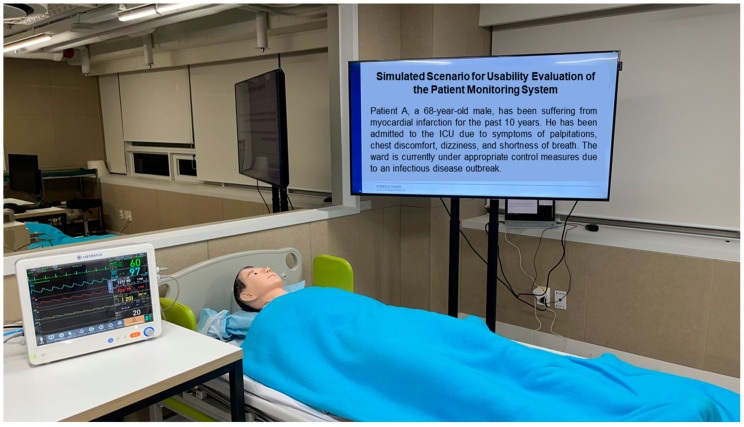
Test environment designed to simulate an intensive care unit (ICU).

The test environment, as depicted in Figure 4, was organized to resemble an intensive care unit using the patient monitoring system. The setup included a patient bed, a patient dummy, and a nursing station, all arranged to closely resemble a real intensive care unit. Temperature and humidity levels in the evaluation room were measured and recorded immediately prior to the evaluation. Consistent with conditions in a typical intensive care unit, the temperature was maintained between 21 °C and 24 °C, and the humidity was kept between 30% and 60%.

**Figure 5:**
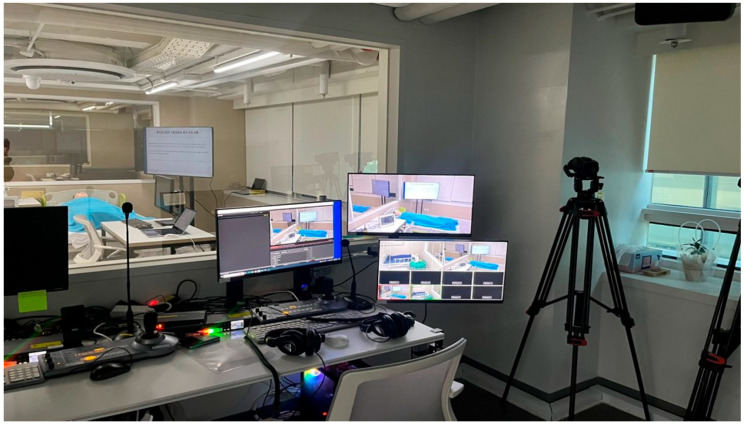
Test observation environment.

The test moderator provided guidance, guiding the participant upon request for assistance with the use scenario, while an observer recorded all participant interactions from outside the test room using a one-way mirror. The setup of the observation environment is illustrated in Figure 5. The observer utilized Blackmagic Media Express software (version 1.0) to document the progress of the usability evaluation.

### 2.3. Statistical Analysis

#### 2.3.1. Satisfaction Survey

A satisfaction questionnaire was designed for each scenario. Participants were asked to rate their experience on a scale from 1 to 5, with 5 representing the highest level of satisfaction and 1 representing the lowest. This questionnaire aimed to collect data on the ease of use for each scenario.

As shown in Table 2, for alarm-related critical tasks, additional satisfaction assessments were conducted beyond just evaluating usefulness. This included evaluating the usefulness of features for adjusting alarm volume and occurrence frequency by priority, as well as assessing the visual and auditory usability of the graphical user interface when different priority alarms were triggered. Usability was evaluated based on five criteria: intuitiveness, visibility, effectiveness, consistency, and simplicity. The principles for evaluating the usability of a design are fundamentally based on Jacob Nielsen’s 10 usability heuristics and Zhang’s 14 heuristic principles [9,32,33,34,35,36]. Subsequently, Steve Krug emphasized the importance of designing interfaces to be as straightforward and easy to use as possible [36,37]. Alison J. Head further advocated for user-centered interfaces, highlighting the importance of task support, usability, aesthetic design, and interface consistency [36,38,39]. These works collectively contributed to the development of usability evaluation principles.

Peter Morville’s research introduced the seven principles of usability: usefulness, usability, value, attractiveness, trustworthiness, discoverability, and accessibility [39,40]. Meanwhile, Donald Norman’s work focused on six critical design principles: visibility, feedback, affordances, mapping, constraints, and consistency [39,41]. These usability indicators have continued to be foundational in the field [39]. Through these foundational studies, five usability evaluation factors were derived by identifying principles emphasized by multiple researchers [39]. Intuitiveness, a principle underscored by Nielsen, Donald Norman, Steve Krug, and Alison J. Head, evaluates whether users can easily locate and understand the desired user interface elements without additional effort. Visibility, highlighted by Nielsen, Zhang, Donald Norman, and Alison J. Head, measures whether users are provided with clear information about their actions, the system’s current state, and the outcomes of those actions. Efficiency, emphasized by Nielsen, Zhang, and Peter Morville, assesses the functional effectiveness of the device, ensuring that users can perform tasks with minimal effort and maximum productivity. Consistency, a key principle advocated by Alison J. Head, ensures that interface elements remain uniform in design and behavior, promoting familiarity and ease of use. Lastly, simplicity, as stressed by Steve Krug and Alison J. Head, evaluates the structural and visual clarity of the device, ensuring that it remains straightforward and easy to use without unnecessary complexity. These factors collectively form a robust framework for usability evaluation, guiding the design and assessment of user interfaces. For this study, the usability principles prioritized were consistently emphasized by at least two researchers. These selected principles align with the requirements of the device design context, providing a robust framework for usability evaluation.

#### 2.3.2. Health-ITUES

Health-ITUES is an evaluation method designed to assess the user interface of medical information technology systems, developed by Po-Yin Yen in 2010 [42,43,44,45]. As detailed in Table 3, Health-ITUES comprises 20 questions distributed across four evaluation areas: impact, perceived usefulness, perceived ease of use, and user control [45]. Participants responded to each question using a 5-point Likert scale, where 1 indicates strong dissatisfaction, and 5 indicates strong satisfaction [45]. Additionally, comprehensive qualitative feedback was collected for each evaluation area to gain deeper insights into user experiences and perceptions. These qualitative data were analyzed thematically to identify common trends and issues highlighted by participants, providing a richer understanding of the strengths and areas for improvement in the user interface. This survey is tailored to enhance usability in the medical field, specifically customized to address medical contexts. It offers the advantage of identifying and addressing usability issues early in the system design and development stages. By evaluating four distinct areas of usability, the measure provides a comprehensive assessment, allowing for an in-depth understanding of how users interact with and perceive the system. Participants’ responses were used to gauge satisfaction levels and identify areas for potential enhancements in the user interface of the medical information technology systems being evaluated.

When comparing the System Usability Scale (SUS) and Health-ITUES, key differences and advantages emerged. The SUS, widely used across various domains, consists of ten items and provides a general assessment of overall usability, making it suitable for a broad range of systems [25,26]. The SUS offers a straightforward measure of overall satisfaction and is known for its simplicity and ease of use. While the SUS provides a single composite score for general usability, Health-ITUES delivers a nuanced analysis through its multi-dimensional framework, which identifies specific areas of strength and improvement. Both tools use a 5-point Likert scale, but Health-ITUES’ detailed and context-specific approach offers deeper insights into user interactions within medical environments, whereas the SUS provides a broader, generalized assessment suitable for diverse systems. The Health-ITUES survey was selected and conducted to gain a more detailed analysis of usability across four specific areas compared to the SUS.

#### 2.3.3. Data Analysis

The satisfaction evaluation and Health-ITUES results were computed using SPSS version 22 (IBM Corp., Armonk, NY, USA) [46]. Participants were compared based on age, affiliation, work experience, and experience with similar devices. Descriptive statistics are presented as means and standard deviations, with significance set at *p* < 0.05. To analyze the eye tracking data, we utilized Tobii Pro Lab for data processing and evaluation.

## 3. Results

### 3.1. User Statistics

A total of 22 participants were recruited from nurses at Severance Hospital (South Korea). Table 4 shows the sociodemographic characteristics of participants. Participants were recruited from Severance Hospital, with ages ranging from 20s to 50s. For nurses, those with different experience levels were recruited, and opinions were collected from all the participants. In particular, those with more experience in using patient monitors were able to gather relevant opinions because they were more familiar with the device, while those with less experience focused on whether the device was easy to use without much experience. We also recruited participants from four different intensive care units: the general ICU, Medical ICU (MICU), Surgical ICU (SICU), and Cardiovascular Care Unit (CCU). This enabled us to evaluate the usability of the patient monitor across each of these departments. The departments in the evaluation primarily used patient monitors from Philips, with GE Healthcare as the secondary provider.

### 3.2. Task Completion

The 22 nurses performed 30 detailed tasks across six extensive scenarios. For usability tests, improvements are made based on analyzing the success rate rather than simply assessing whether a predefined target has been met. Understanding the reasons behind a low success rate is prioritized. Achieving a specific target value is not necessary for an evaluation to be deemed successful, as such target values are generally determined based on the company’s internal standards. As shown in Table 5, participants achieved a success rate of 70% or higher in all six scenarios, with the lowest success rate being 78.80% in the scenario involving a data trend review.

Among the scenarios where the participants did not successfully complete a task during the evaluation, the following critical task in the alarm scenario was identified: Task 25, “Confirm that the current ECG waveform is normal and tap the message list to view and clear any arrhythmia alarms that have occurred to date”. Participants were able to observe the list of messages but did not recognize that the “Audio Alarm Pause” button at the bottom of the screen was intended for clearing messages. Consequently, participants attempted to delete messages by clicking directly on them, resulting in a failure to complete the task.

Although not considered high-severity tasks, many participants struggled with completing tasks in both the remote control scenario and the data trend review scenario. There are four tasks with a success rate below 80%, one of which is a critical task, while the remaining three are non-critical tasks. In Task 2, participants attempted to verify if the remote control feature was enabled but failed to locate the icon at the top of the screen that indicated the feature’s activation. Additionally, in Task 27, related to the event review screen, nine participants were unable to access the menu. In existing patient monitoring systems with prior experience, a review button is present at the bottom of the main screen, facilitating easy access. However, the evaluation device lacked a review button on the main screen, which contributed to a lower success rate for the task. In Task 29, five participants were unable to complete the task. This failure occurred for several reasons: some participants mistakenly pressed the “Alarm Pause” button or accessed the event history, while others struggled with the task due to a lack of understanding of the procedure for data deletion. Specifically, participants had difficulty identifying and pressing the “Setting” button within the event review window and subsequently failed to delete the data using the “Clear Event” button.

### 3.3. Eye Tracking Analysis to Usability Test Results

**Figure 6:**
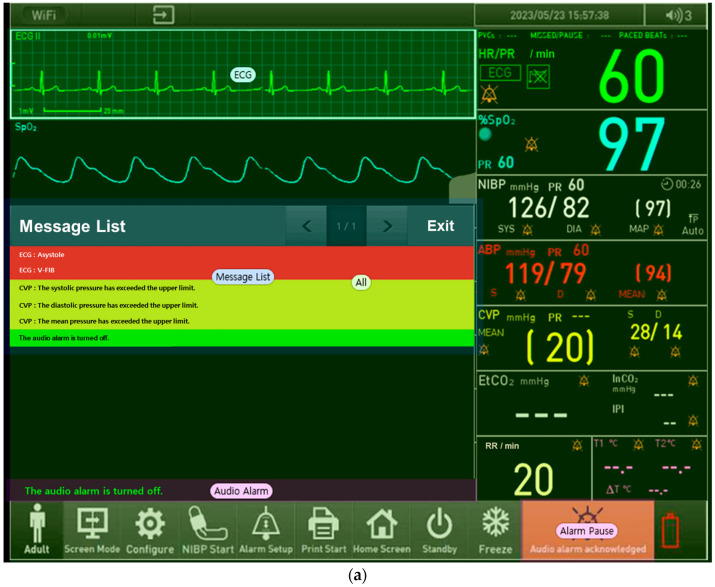

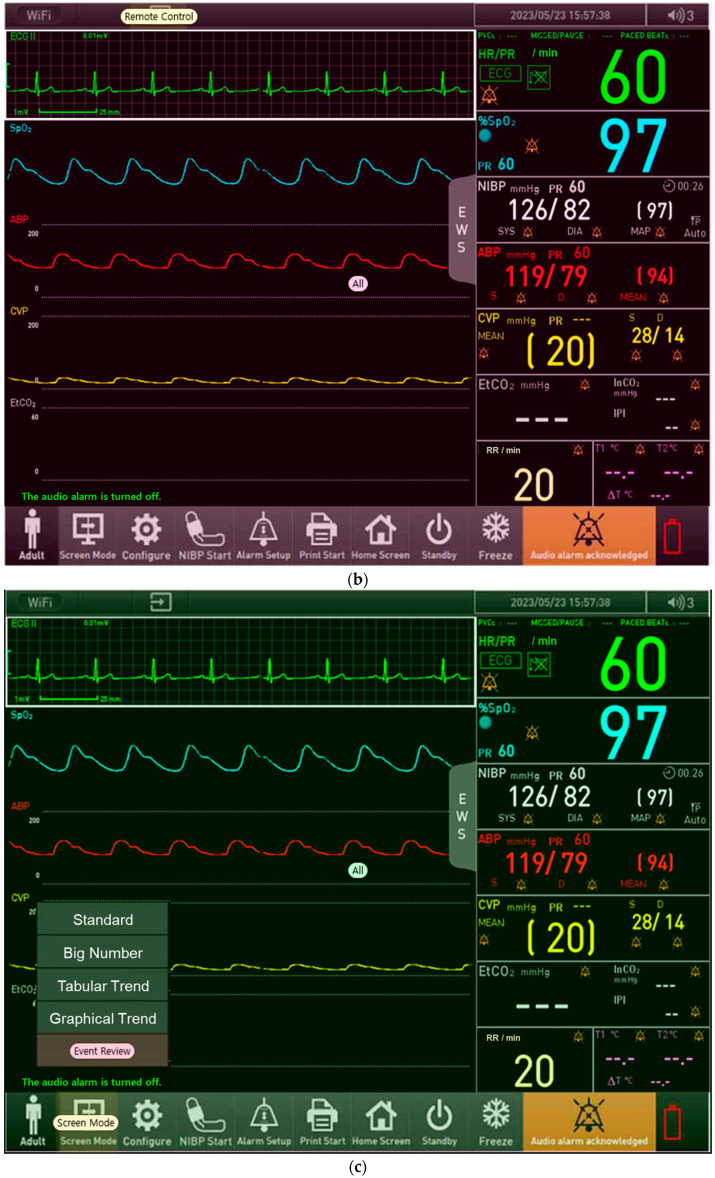

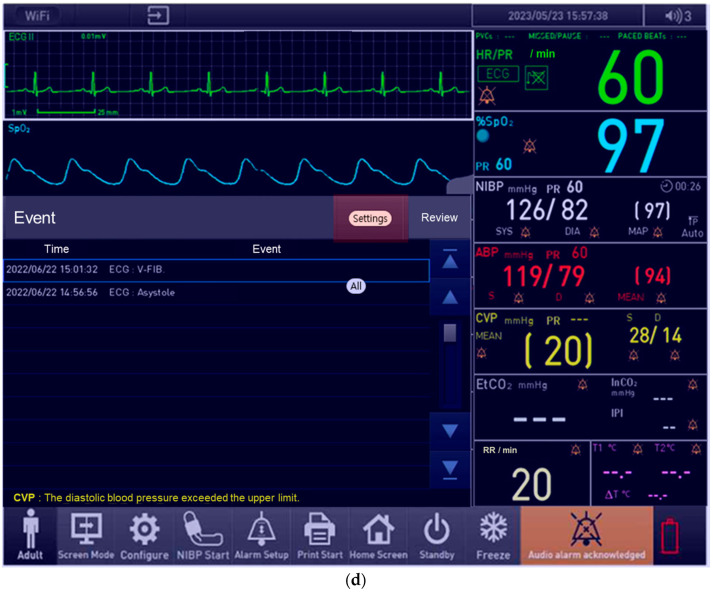
Representative AOI images: (**a**) the eye-tracking image of a representative user who failed Task 25, (**b**) the eye-tracking image of a representative user who failed Task 2, (**c**) the eye-tracking image of a representative user who failed Task 27, and (**d**) the eye-tracking image of a representative user who failed Task 29.

We conducted a detailed analysis of participants’ gaze patterns for tasks with low success rates using eye-tracking technology. To identify the areas where participants should focus their gaze during task performance, areas of interest (AOIs) were defined, as shown in Figure 6. For all four tasks, AOIs were commonly divided into two categories: the buttons participants needed to focus on and all other background areas excluding the buttons.

For Task 25, participants needed to confirm that the current ECG waveform was normal, check the arrhythmia alarms in the message list, and then delete them. This task required four distinct actions: first, confirming the ECG waveform in the AOI located at the topmost area of the screen; second, selecting the AOI for the audio alarm, followed by reviewing the message list; and, finally, focusing on the AOI for the alarm pause to touch and delete the alarm. For Task 2, the AOI was set to the icon indicating whether the remote control feature was active, with the rest of the screen designated as a background AOI. In Task 27, three AOIs were defined: the “Screen Mode” button located at the bottom of the screen, the “Event Review” button accessible after pressing “Screen Mode”, and the remaining background. For Task 29, the AOIs were designated as the “Settings” button located in the center of the screen and the remaining background.

These AOI definitions provided a structured basis for analyzing participants’ gaze behavior in each task.

**Figure 7:**
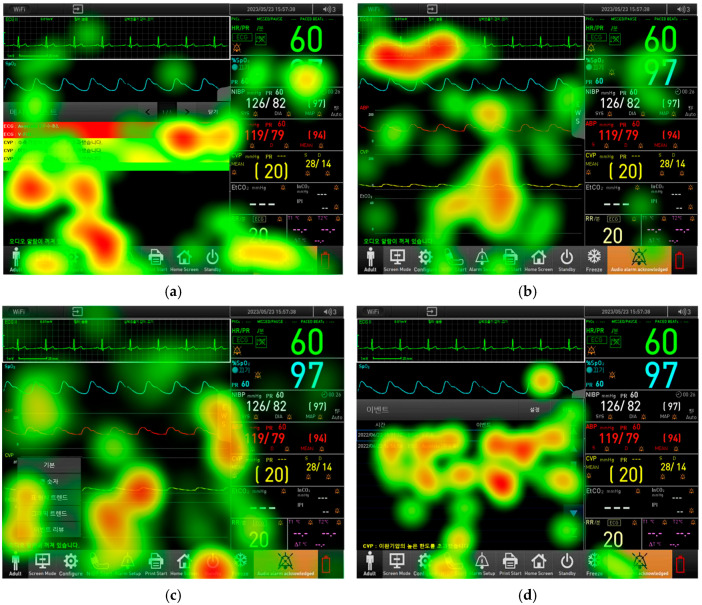
Representative heatmap images: (**a**) the eye-tracking image of a representative user who failed Task 25, (**b**) the eye-tracking image of a representative user who failed Task 2, (**c**) the eye-tracking image of a representative user who failed Task 27, and (**d**) the eye-tracking image of a representative user who failed Task 29. Green to red color represents fixation value level where green stands for lowest value and red represents highest value. Refer to Figure 6 for representative AOI for heatmap.

Figure 7 portrays representative gaze pattern data shown in heatmap form for low success tasks. Figure 7a presents the gaze data for Task 25, which was a critical task. Our analysis revealed that participants who failed this task predominantly focused on the alarm messages within the message list window. Despite the presence of the “Alarm Pause” button, they did not recognize the option to delete the arrhythmia alarm, as evidenced by the heatmap.

Figure 7b shows the gaze patterns for Task 2, which, although not critical, had a low success rate. Participants did not attend to the icon at the top of the screen while examining waveforms or parameters, leading them to overlook whether the remote control feature was activated.

Figure 7c illustrates the gaze patterns for Task 27. Participants in this task often fixated on the menu button or explored the screen mode options in the menu bar but struggled to enter the event review window. They frequently lingered on the position of the alarm message list.

Finally, Figure 7d depicts the gaze patterns for Task 29. To delete event history, participants displayed a gaze pattern similar to that in Task 25, focusing on the “Pause Alarm” button rather than the actual event history deletion option within the settings menu. Many participants neglected to direct their gaze toward the settings, often opting instead to interact with the review button adjacent to the settings.

**Figure 8:**
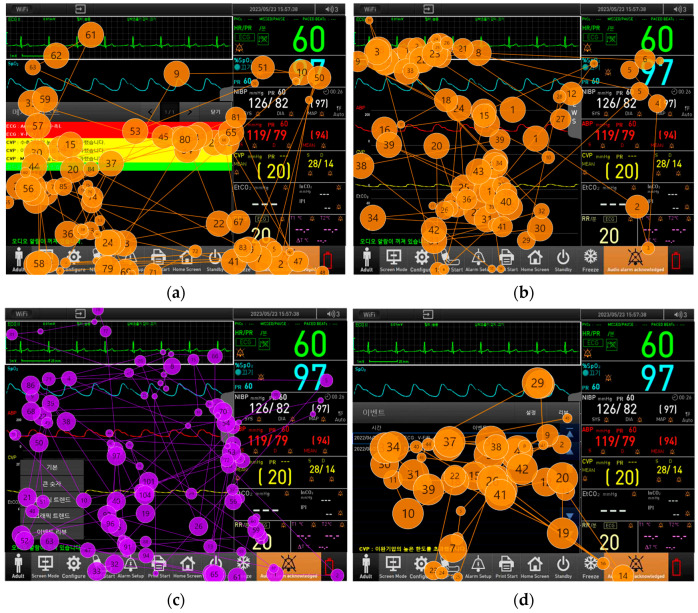
Representative gaze plot images: (**a**) the eye-tracking image of a representative user who failed Task 25, (**b**) the eye-tracking image of a representative user who failed Task 2, (**c**) the eye-tracking image of a representative user who failed Task 27, and (**d**) the eye-tracking image of a representative user who failed Task 29. The number sequence represents the start and end of gaze within the following tasks.

Figure 8 illustrates gaze plots for tasks with low success rates, similar to Figure 7. Gaze plots represent the sequence of gaze movement using circles and connecting lines, where the size of the circles indicates the duration of gaze fixation.

For Tasks 25, 27, and 29, participants’ gaze lingered on areas related to the intended task, yet they failed to identify the corresponding buttons and moved on. In contrast, for Task 2, it was observed that participants did not direct their gaze to the relevant areas at all.

### 3.4. Usability (Satisfaction Survey)

The satisfaction scores for each scenario item were all four points or higher, with the highest satisfaction reported for the data trend review item, as shown in Table 6. Participants indicated that the ability to review missed event details was particularly useful and helpful. In contrast, the remote control function achieved a target score of 3.5 or higher but received lower satisfaction compared to other items. This lower satisfaction may be attributed to the nature of the intensive care unit as a restricted area, where most device access is limited to medical staff. Consequently, some participants felt that a password was unnecessary. However, it should be noted that, since 2019, Korea’s Ministry of Food and Drug Safety has mandated cybersecurity requirements for medical device software, including password protection for devices used in restricted areas. The lower satisfaction may reflect participants’ familiarity with older medical devices that predate these cybersecurity mandates.

The activation of the remote control function in Task 2 recorded the lowest task success rate at 59.09%, and the satisfaction score for this function was 3.86 (0.81). This indicates that many users experienced difficulties in performing this task, with the satisfaction level also being the lowest among all evaluated features. Specifically, nurses faced challenges in activating or verifying the activation of the remote control function, largely due to the unfamiliarity with this feature, as it was not present in previously used devices. This unfamiliarity is likely a key factor contributing to the lower satisfaction scores. Tasks 27 and 29, both related to event review, showed task success rates of 59.09% and 77.27%, respectively. The satisfaction score for these tasks was 4.68 (0.55), indicating a relatively high level of satisfaction. Although users encountered some difficulties in locating the event review function, once they identified its position within the interface, they found it easy to access. This ease of access after initial identification likely contributed to the relatively high satisfaction score.

In the satisfaction survey related to alarm items, a detailed analysis of the critical tasks revealed that the feature for adjusting alarm volume by priority received a score of 4.27 (0.86), while the feature for adjusting alarm occurrence frequency by priority received a score of 3.97 (1.04). Alarms are categorized by risk levels—high, mid, and low. To assess the effectiveness of visual and auditory user interfaces in distinguishing these risk levels, a survey was conducted using five different criteria, as shown in Table 7. The usability score for the risk level-based user interface was 4.15 (0.88). However, some aspects scored lower than the average, particularly in terms of effectiveness, consistency, and simplicity. It was noted that the visual indicators for mid and low-priority alarms, which were both represented in yellow, made it difficult to differentiate between them. Additionally, the auditory signal for high-priority alarms was reported to be challenging to perceive during emergencies.

### 3.5. Usability (Health-ITUES)

The Health-ITUES satisfaction evaluation demonstrated high levels of satisfaction, with scores of 4.45 in impact and 4.25 in perceived usefulness, as shown in Table 8. Participants frequently noted that the system’s array of functions would enhance the efficiency of monitoring patient vital signs. Furthermore, the capability to set the high alarm volume to be louder than other alarms was identified as an effective feature for managing various risk levels. The Health-ITUES survey revealed that the question concerning user control, specifically regarding the ease and speed of correcting mistakes, received the lowest score, which was 3.43 (0.89).

In addition, learning how to operate a new device is crucial for effective use. In the perceived ease of use category, the item “The system is easy to learn” received a score of 3.9 (0.79). However, the item “It will be easy to become skillful at using the system” scored higher at 4.14 (0.87), indicating that, while initial learning may be challenging, users are likely to adapt and become proficient with continued use. Participants in the evaluation noted that, although the device was simple and intuitive, they experienced some difficulty due to unfamiliarity with the new system. Nevertheless, they believed that, after receiving training, they would be able to use it more easily, and they anticipated that they would complete tasks more quickly in future evaluations.

## 4. Discussion

This study involved conducting usability tests and surveys with 22 nurses from various intensive care units (ICUs) who primarily use patient monitoring systems. To gain a more detailed understanding of usability, we also used eye-tracking technology to capture the gaze pattern of each participant.

The evaluation results showed that participants achieved a minimum success rate of 78% in usability tests and reported a satisfaction score of at least four out of five.

When correlating the usability test results with eye tracking data, it became apparent that failures in task performance were generally due to either a lack of gaze fixation on the relevant features or an inability to recognize the function of the features even when gaze fixation occurred. For Task 25, the gaze plot indicated that participants frequently directed their gaze toward the message list and the alarm pause button but moved their gaze towards other sections of the monitor. This indicates that, even though their gaze was upon the message list and the alarm pause button, they did not recognize the function of the button, leading to incompletion of the task. Specifically, for Task 25 and Task 29, both of which involved deletion tasks, there were notable differences in the deletion methods: Task 25 required the deletion of alarm messages, while Task 29 involved deleting event history. Eye tracking results revealed that participants in Task 29 frequently focused on the “Alarm Pause” button, which is intended to pause ongoing alarms rather than delete them. This confusion was evident as participants repeatedly clicked and gazed at the message areas in both Task 25 and Task 29. In Task 25, alarms with lower or intermediate priority could be deleted by clicking the alarm message, whereas high-priority alarms required the “Alarm Pause” button to be pressed. This discrepancy led to considerable user confusion. For Task 29, Figure 7d and Figure 8d show that participants fixated on the “Alarm Pause” button twice, with much of their gaze directed toward the descriptions of the events that had occurred. During the task of deleting the event history, participants were expected to focus on the “Settings” area; however, it was observed that their gaze never reached the “Settings” button.

Furthermore, the user interface of the device differed from that of other systems previously used by participants, where alarm deletion or acknowledgment was more straightforward and integrated into the main screen. The absence of a dedicated delete button and reliance on the “Alarm Pause” button, which serves dual functions—pausing alarms and deleting high-priority alarms—resulted in poor usability and task failure. To address these issues, it is recommended that the “Alarm Pause” button be limited to only pausing alarms, allowing users to complete patient care or acknowledge alarms without additional functionality. Additionally, incorporating a dedicated delete button within the alarm message list would provide a more intuitive means of deleting alarms. Adding such intuitive buttons to the interface would enhance both usability and visibility for users.

In Task 2, which involves verifying whether the remote control feature is activated, both eye tracking and usability testing revealed that participants often did not recognize whether the icon indicated activation. An analysis of the heatmap and gaze plot in Figure 7b and Figure 8b revealed that participants did not direct their gaze toward the remote control icon, nor was any gaze detected near it. Instead, participants primarily focused on the waveform area. The absence of gaze toward the icon suggests a lack of intuitive design, indicating that participants found it difficult to recognize the icon as a means of confirming whether the remote control feature was activated. This issue was particularly pronounced in the intensive care units of hospitals, where the remote control feature is essential. Many participants questioned the necessity of the icon, reflecting uncertainty about its role. Additionally, unlike other devices where the central monitoring system displays patients’ vital signs when connected, participants were unfamiliar with this feature, leading to a lower task success rate. The satisfaction score for this task was also the lowest among all items, at 4.04 (0.89). This indicates a correlation between task failure rates and satisfaction scores for this item. To address this, emphasizing the importance of the remote control feature in patient monitoring devices and providing thorough training to medical staff on the use of this icon could help improve both task success rates and user satisfaction.

For Task 27, although the task success rate was relatively low at 59.09%, the satisfaction score was high, at 4.68 (0.55). This indicates that, while the task success rate was suboptimal, participants reported high satisfaction with the overall user interface. The eye-tracking results revealed that participants did not recognize the method for accessing the event review through the screen mode button. Participants briefly looked at the event review area after pressing the screen mode button but did not seem to recognize its functionality. As shown in Figure 8c, the gaze plot revealed that participants fixated on the Event Review menu tab three times but still failed to locate it. Instead, their gaze was primarily directed toward the EWS (Early Warning Score) menu tab. Unlike the Event Review menu, the EWS menu is designed to predict and identify potential risks by analyzing parameter values in advance. Discussions with healthcare professionals regarding the tendency to focus on the EWS menu revealed that the Event Review menu was not visible on the main screen. Additionally, the proximity of the EWS menu to the waveform area led participants to mistakenly perceive it as the tab for reviewing events. This discrepancy suggests that, while users were generally satisfied with the event review window itself, the method for accessing it differed from other devices they had used, contributing to the lower task success rate. To improve usability and accessibility, it is recommended to add a review button to the bottom menu of the main screen. This change would enhance accessibility and streamline the user experience, particularly in intensive care units where the continuous monitoring of critically ill patients is essential. Given the need for frequent and quick access to patient status trends, having a review button readily available on the main screen would facilitate easier and faster access.

In the Health-ITUES survey, the scores for perceived ease of use and satisfaction with one’s ability to use the device were 3.81 (0.83) and 3.57 (0.72), respectively. Additionally, for the user control item, which assesses whether users can easily and quickly correct mistakes made while using the device, the score was 3.43 (0.89). All three scores were below the overall average score of 4.13 (0.78). Analysis of the usability test and eye tracking data revealed that participants with lower task success rates also reported lower scores on perceived ease of use. Eye tracking data showed that these participants often failed to view the appropriate screens necessary for task completion. The correlation between these three items indicates that issues with perceived ease of use and user control were associated with task performance. Furthermore, the user control item, which had the lowest satisfaction score, highlighted a lack of guidance when errors occurred during task performance. In usability tests, participants who encountered errors often had no on-screen prompts and needed to request assistance from the facilitator. Eye tracking results showed that, when participants could not find the correct function due to the absence of guidance messages, they ended up repeatedly searching different areas of the screen, leading to task failure.

A limitation of this study is that the usability testing was conducted using an eyeglasses-type eye tracker, which was not usable for individuals who wore glasses. Despite this constraint, it is important to note that this issue does not impact the overall validity of the usability assessments. This study evaluated 22 nurses from Severance Hospital in Korea; thus, the findings may reflect the opinions of nurses from a single institution. In future research, we plan to conduct evaluations with nurses from various hospitals and include only those who do not wear glasses. A notable strength of this study is the inclusion of a diverse cohort of ICU nurses who regularly use the device, ensuring that the findings are representative of actual user experiences in a relevant clinical context.

In previous studies, eye-tracking technology has been utilized as a supplementary method to objectively evaluate participants’ performance during usability tests [24]. Two key evaluation metrics were employed to assess the user interface: changes in pupil diameter and the slope of pupil diameter change over time [24]. Pupil diameter serves as a sensitive indicator of task difficulty and cognitive demands [24].

Building on these findings, future studies will include additional analyses of pupil diameter changes and the slope of these changes over time to investigate their correlation with task completion and duration [24]. This approach aims to provide deeper insights into the relationship between cognitive workload and performance efficiency during usability evaluations.

## 5. Conclusions

We recruited 22 nurses working in intensive care units and conducted usability testing and surveys in an evaluation environment mimicking the ICU at the Medical Device Usability Research Center, Gangnam Severance Hospital. This study aimed to identify use errors and usability issues with the medical device. Participants achieved an average task success rate of 94% and reported a satisfaction score of 4.24. The Health-ITUES survey yielded a score of 4.13.

The analysis of use errors leveraged data from eye tracking, survey results, subjective feedback, and objective gaze observations to identify the root causes of errors. One notable finding was that participants, as first-time users of the device, initially struggled with the unfamiliar screen layout, which led to recurring errors. However, it is anticipated that usability will improve with targeted training and increased user experience.

In future research, we plan to strengthen the analytical capabilities of eye tracking data by incorporating metrics such as fixation and saccade, which were not utilized in this study. Additionally, we aim to refine data interpretation through advanced quantitative methods, such as statistical analysis, to identify subtle patterns in user behavior. Furthermore, we intend to expand the scope of application to various clinical settings and user groups, providing more comprehensive insights. Integrating these advancements into usability evaluations will allow us to derive more comprehensive insights for optimizing user interface design. These efforts will ultimately contribute to optimizing user interfaces and addressing usability challenges more effectively.

To further assess suitability for use and clinical effectiveness, it is essential to evaluate how effectively a monitoring system directs clinicians’ attention to critical patient-related information [47]. Eye tracking data can be utilized to analyze the effectiveness of visual signals, such as color changes and flashing notifications, in guiding attention. Previous studies have demonstrated that social stimuli, such as gaze direction and facial expressions, significantly influence visual attention [47]. These principles can be integrated into the design of patient monitoring systems.

In particular, incorporating visual cues that simulate social attention dynamics could help direct clinicians’ focus to key areas relevant to patient care. This study proposes to evaluate whether such design elements effectively direct attention in real-world settings, contributing to the overall suitability and clinical effectiveness of monitoring systems. By emphasizing these aspects, we aim to enhance the usability and functionality of medical devices in critical care environments.

## Figures and Tables

**Table 1:** Use scenarios for evaluating the usability of patient monitoring systems.

Use Scenario	No.	Task
Remote Control	Task 1	Turn the remote control to On.
	Task 2	Make sure the remote control is activated.
Registering Patients	Task 3	Admit a new patient.
	Task 4	Check the registered patient’s information in the central monitoring system.
Connecting Cables	Task 5	Read the manual to learn about device drop precautions.
	Task 6	Connect the SpO_2_ cable.
	Task 7	Connect the NIBP cable.
	Task 8	Connect the Temperature cable.
	Task 9	Randomly change the NIBP measurement interval and verify that it is reflected in the central monitoring system.
	Task 10	Measure NIBP and verify the measurement in the CMS.
Alarm	Task 11	Select ‘User Configuration Mode’.
	Task 12	Set the volume of the high alarm to be louder than the other alarm volumes.
	Task 13	Change the audible alarm interval for the low alarm to 30 s.
	Task 14	Make the alarm limits changeable.
	Task 15	Make the alarm limits visible on the monitor, and point to the location where the alarm limits are displayed.
	Task 16	Point directly to visual alarm (SpO_2_) location for confirmation.
	Task 17	Pause the audible alarm that is currently occurring.
	Task 18	Confirm that the audible alarm pause has been applied to the CMS.
	Task 19	Change the alarm lower limit value for SpO_2_ to 85%.
	Task 20	Confirm in the CMS that the alarm lower limit value for SpO_2_ has changed.
	Task 21	Change the settings so that all types of arrhythmia alarms can occur.
	Task 22	Change the alarm condition for Ventricular Tachycardia to 135 bpm.
	Task 23	Check the visual and audible cues for a medium-risk alarm (Tachycardia).
	Task 24	Verify the visual and audible cues for the high-risk V-FIB alarm, and indicate the area where the visual cues and alarm messages are occurring.
	Task 25	Confirm that the current ECG waveform is normal, and tap the message list to view and clear any arrhythmia alarms that have occurred to date.
	Task 26	Check for SpO_2_-related alarm messages and reconnect the SpO_2_ cable.
Data Trend Review	Task 27	Confirm the Event Review window on the screen.
	Task 28	Review the details of the V-FIB event that occurred.
	Task 29	Delete the event history.
Standby Mode	Task 30	Switch to Standby Mode.

**Table 2:** Satisfaction survey items for each function of the patient monitoring device.

ITEMS	Statements
Remote control	Do you consider the remote control function easy to use?
Registering patients	Do you consider the patient registration and discharge process easy to use?
Connecting cables	Do you consider the cable connection process and method easy to use?
Alarm	Do you consider the alarm function easy to use?
	User config menu—Do you consider the feature for adjusting the alarm volume by priority easy to use?
	User config menu—Do you consider the feature for adjusting the alarm occurrence frequency by priority useful?
	Graphical user interface—Intuitiveness
	Graphical user interface—Visibility
	Graphical user interface—Effectiveness
	Graphical user interface—Consistency
	Graphical user interface—Simplicity
Data trend review	Do you consider the event review function easy to use?
Standby mode	Do you consider the standby mode function easy to use?

**Table 3:** Health-ITUES Items.

ITEMS	Statements
Impact	I think M50 would be a helpful device for persons who monitor patients.
	I think M50 would improve the quality of life for the users who monitor patients.
	M50 would be an important part of the intensive care unit where patients are hospitalized.
Perceived Usefulness	Using M50 makes it easier to monitor patients.
	Using M50 enables users to easily understand the patient’s condition.
	Using M50 makes it more likely that users can respond to patient emergencies.
	Using M50 is useful for managing patient health conditions.
	I think the M50 provides a faster process for managing patient health.
	I am satisfied with the M50 for managing patient conditions smoothly.
	The M50 has enabled me to respond to patient alarms in a timely manner.
	Using the M50 improves my ability to monitor patients.
	I can make the appropriate settings for patient monitoring every time I use the M50.
Perceived Ease of Use	I am comfortable with my ability to use the M50.
	Learning to operate M50 is easy for users.
	It is easy for users to become skillful with the M50.
	I find the M50 easy to use.
	I can always remember how to manage patients using the M50.
User Control	M50 gives error messages that clearly tell users how to fix the problem.
	Whenever I make a mistake while using M50, I can fix it quickly and easily.
	The information (such as online help, on-screen messages, and other documentation) provided with M50 is clear.

**Table 4:** Sociodemographic characteristics and experience of the test participants.

Variable	Option	Frequency
Age	20–29 years	6
	30–39 years	9
	40–49 years	5
	50–59 years	2
Affiliated hospital of participants	Severance Hospital	22
Department of participants	Intensive Care Unit	11
	Medical Intensive Care Unit (MICU)	3
	Surgical Intensive Care Unit (SICU)	1
	Cardiac Care Unit (CCU)	7
Work experience	Less than 5 years	3
	More than 5 years, less than 10 years	10
	More than 10 years	9
Manufacturer name	GE	7
	Phillips	16
	Drager	1
	Mediana	1
Use experience with similar devices	Less than 3 years	1
	More than 3 years, less than 5 years	3
	More than 5 years, less than 10 years	9
	More than 10 years	9

**Table 5:** Task completion rate.

Category	Task Pass Rate (%)	Task Failure Rate (%)
Remote control	79.50	20.50
Registering patients	100	0.00
Connecting cables	97.00	3.00
Alarm	89.80	10.20
Data trend review	78.80	21.20
Standby mode	100	0.00

**Table 6:** Satisfaction scores for the user interface of the patient monitoring systems.

Category	Mean	SD
Remote control	4.04	0.89
Registering patients	4.59	0.78
Connecting cables	4.30	0.78
Alarm	4.10	0.92
Data trend review	4.68	0.55
Standby mode	5.00	0.00

**Table 7:** Usability scores for the alarm user interface of patient monitoring systems.

Category	Mean	SD
Intuitiveness	4.36	0.88
Visibility	4.32	0.70
Effectiveness	4.00	0.95
Consistency	4.05	0.88
Simplicity	4.00	1.00

**Table 8:** Descriptive statistics: scale scores for the Health-ITUES subscales.

Category	Mean	SD
Impact	4.45	0.67
Perceived usefulness	4.25	0.78
Perceived ease of use	3.85	0.82
User control	3.95	0.84
Overall Health-ITUES score	4.13	0.78

## Data Availability

Data are available upon demand.

## References

[1] Andrade E., Quinlan L., Harte R., Byrne D., Fallon E., Kelly M., Casey S., Kirrane F., O’Connor P., O’Hora D. (2020). Novel interface designs for patient monitoring applications in critical care medicine: Human factors review. JMIR Hum. Factors.

[2] Walsh T., Beatty P.C. (2002). Human factors error and patient monitoring. Physiol. Meas..

[3] Gardner R.M., Clemmer T.P., Evans R.S., Mark R.G. (2014). Patient monitoring systems. Biomedical Informatics: Computer Applications in Health Care and Biomedicine.

[4] Baig M.M., GholamHosseini H., Moqeem A.A., Mirza F., Lindén M. (2017). A systematic review of wearable patient monitoring systems–current challenges and opportunities for clinical adoption. J. Med. Syst..

[5] Fidler R., Bond R., Finlay D., Guldenring D., Gallagher A., Pelter M., Drew B., Hu X. (2015). Human factors approach to evaluate the user interface of physiologic monitoring. J. Electrocardiol..

[6] Li H., Ku M., Schumacher R., Seagull F.J. (2013). Designing automated aids for patient monitoring systems in intensive care units. Proceedings of the International Symposium on Human Factors and Ergonomics in Health Care.

[7] Martin J.L., Norris B.J., Murphy E., Crowe J.A. (2008). Medical device development: The challenge for ergonomics. Appl. Ergon..

[8] Food and Drug Administration (2016). Applying human factors and usability engineering to medical devices: Guidance for industry and Food and Drug Administration staff. Fed. Regist./FIND.

[9] Ribeiro Custódio R.A., Trzesniak C., Pinto Ribeiro Miranda R., Donda Angelini G.H., Bordon J.S., Santos Vieira L.C., Pereira Mello C.H. (2019). Applying human factors engineering methods for risk assessment of a neonatal incubator. J. Healthc. Eng..

[10] (2016). Medical Devices–Part 2: Guidance on the Application of Usability Engineering to Medical Devices.

[11] (2004). Medical Electrical Equipment, Part.

[12] (2015). 2015: Medical Devices–Part 1: Application of Usability Engineering to Medical Devices.

[13] FDA (2022). Content of Human Factors Information in Medical Device Marketing Submissions.

[14] Hilgers J.E., Ten Brink K.M. (2018). Assessing Medical Device Usability with Eye-Tracking. Proceedings of the International Symposium on Human Factors and Ergonomics in Health Care.

[15] Holmqvist K., Nyström M., Andersson R., Dewhurst R., Jarodzka H., Van de Weijer J. (2011). Eye Tracking: A Comprehensive Guide to Methods and Measures.

[16] Koester T., Brøsted J.E., Jakobsen J.J., Malmros H.P., Andreasen N.K. (2017). The use of eye-tracking in usability testing of medical devices. Proceedings of the International Symposium on Human Factors and Ergonomics in Health Care.

[17] Krafka K., Khosla A., Kellnhofer P., Kannan H., Bhandarkar S., Matusik W., Torralba A. Eye tracking for everyone. Proceedings of the IEEE Conference on Computer Vision and Pattern Recognition.

[18] Pauszek J.R. (2023). An introduction to eye tracking in human factors healthcare research and medical device testing. Hum. Factors Healthc..

[19] Hofmaenner D.A., Herling A., Klinzing S., Wegner S., Lohmeyer Q., Schuepbach R.A., Buehler P.K. (2021). Use of eye tracking in analyzing distribution of visual attention among critical care nurses in daily professional life: An observational study. J. Clin. Monit. Comput..

[20] Jankowska D.M., Czerwonka M., Lebuda I., Karwowski M. (2018). Exploring the creative process: Integrating psychometric and eye-tracking approaches. Front. Psychol..

[21] Mazidi M., Dehghani M., Sharpe L., Dolatshahi B., Ranjbar S., Khatibi A. (2021). Time course of attentional bias to painful facial expressions and the moderating role of attentional control: An eye-tracking study. Br. J. Pain.

[22] Pasarica A., Bozomitu R.G., Costin H., Miron C., Rotariu C. (2017). Human-computer interface based on eye tracking with dwell time selection. Proceedings of the 2017 IEEE 23rd International Symposium for Design and Technology in Electronic Packaging (SIITME).

[23] Vansteenkiste P., Cardon G., Philippaerts R., Lenoir M. (2015). Measuring dwell time percentage from head-mounted eye-tracking data–comparison of a frame-by-frame and a fixation-by-fixation analysis. Ergonomics.

[24] Jiang M., Liu S., Feng Q., Gao J., Zhang Q. (2018). Usability study of the user-interface of intensive care ventilators based on user test and eye-tracking signals. Med. Sci. Monit. Int. Med. J. Exp. Clin. Res..

[25] Cai B., Xu N., Duan S., Yi J., Bay B.H., Shen F., Hu N., Zhang P., Chen J., Chen C. (2022). Eye tracking metrics of orthopedic surgeons with different competency levels who practice simulation-based hip arthroscopic procedures. Heliyon.

[26] Lohmeyer Q., Schiess C., Garcia P.D.W., Petry H., Strauch E., Dietsche A., Schuepbach R.A., Buehler P.K., Hofmaenner D.A. (2023). Effects of tall man lettering on the visual behaviour of critical care nurses while identifying syringe drug labels: A randomised in situ simulation. BMJ Qual. Saf..

[27] Tobii AB (2024). Tobii Pro Glasses 3 User Manual. https://www.tobii.com/products/eye-trackers/wearables/tobii-pro-glasses-3.

[28] Kules B., Xie B. (2011). Older adults searching for health information in MedlinePlus—An exploratory study of faceted online search interfaces. Proc. Am. Soc. Inf. Sci. Technol..

[29] Liu C.-J., Kemper S., McDowd J. (2009). The use of illustration to improve older adults’ comprehension of health-related information: Is it helpful?. Patient Educ. Couns..

[30] Jiang M., Sun D., Li Q., Wang D. (2020). The usability of ventilator maintenance user interface: A comparative evaluation of user task performance, workload, and user experience. Sci. Prog..

[31] Loh Z., Hall E.H., Cronin D., Henderson J.M. (2023). Working memory control predicts fixation duration in scene-viewing. Psychol. Res..

[32] Zhang J., Johnson T.R., Patel V.L., Paige D.L., Kubose T. (2003). Using usability heuristics to evaluate patient safety of medical devices. J. Biomed. Inform..

[33] Nielsen J. Finding usability problems through heuristic evaluation. Proceedings of the SIGCHI Conference on Human Factors in Computing Systems.

[34] Nielsen J. Usability inspection methods. Proceedings of the Conference Companion on Human Factors in Computing Systems.

[35] Nielsen J., Molich R. Heuristic evaluation of user interfaces. Proceedings of the SIGCHI Conference on Human Factors in Computing Systems.

[36] Young C.H., Sung S.J. (2016). A Study on Mobile Application UI Design for Omni-Channel Shopping. J. Korean Soc. Des. Cult..

[37] Krug S. (2000). Don’t Make Me Think! A Common Sense Approach to Web Usability.

[38] Head A.J. (1999). Design Wise: A Guide for Evaluating the Interface Design of Information Resources.

[39] Kim J.H., Lee H.S. (2023). Analysis of Usability Evaluation Factors of Mobile GUI by AHP. J. Korea Des. Forum.

[40] Morville P. (2005). Ambient Findability: What We Find Changes Who We Become.

[41] Tenner E. (2015). The design of everyday things by Donald Norman. Technol. Cult..

[42] Schnall R., Cho H., Liu J. (2018). Health Information Technology Usability Evaluation Scale (Health-ITUES) for usability assessment of mobile health technology: Validation study. JMIR Mhealth Uhealth.

[43] Stonbraker S., Cho H., Hermosi G., Pichon A., Schnall R. (2018). Usability testing of a mHealth app to support self-management of HIV-associated non-AIDS related symptoms. Nursing Informatics 2018.

[44] Loh K.P., Liu J., Ganzhorn S., Sanabria G., Schnall R. (2022). Establishing a usability cut-point for the health information technology usability evaluation scale (Health-ITUES). Int. J. Med. Inform..

[45] Yen P.Y., Wantland D., Bakken S. (2010). Development of a Customizable Health IT Usability Evaluation Scale. AMIA Annu. Symp. Proc..

[46] Chaniaud N., Métayer N., Megalakaki O., Loup-Escande E. (2020). Effect of prior health knowledge on the usability of two home medical devices: Usability study. JMIR Mhealth Uhealth.

[47] Dalmaso M. (2022). Exploring the social environment with the eyes: A review of the impact of facial stimuli on saccadic trajectories. Int. J. Environ. Res. Public Health.

